# Protein misfolding‐specific epitope identification for passive and active immunotherapy of neurodegenerative diseases

**DOI:** 10.1002/alz70859_098670

**Published:** 2025-12-25

**Authors:** Neil R Cashman, Steven S Plotkin, Scott Napper, Ebrima Gibbs, Beibei Zhao, Erin Scruten, Juliane Coutts, Johanne Kaplan

**Affiliations:** ^1^ University of British Columbia, Vancouver, BC Canada; ^2^ ProMIS Neurosciences, Cambridge, MA USA; ^3^ University of Saskatchewan, VIDO, Saskatoon, SK Canada; ^4^ ProMIS Neurosciences, Toronto, ON Canada

## Abstract

**Background:**

Toxic misfolded proteins underlie the pathogenesis of neurodegenerative diseases such as Alzheimer’s and Parkinson’s disease (AD&PD), and amyotrophic lateral sclerosis/frontotemporal dementia (ALS/FTD). Generation of therapeutic antibodies selectively targeting only disease‐misfolded isoforms, while sparing normal or irrelevant isoforms, has not yet been successfully achieved by conventional immunization strategies.

**Method:**

ProMIS Neurosciences has developed a computational platform to identify conformational epitopes that are uniquely exposed on toxic misfolded proteins, which can then be used to generate misfolding‐specific antibodies or vaccine formulations.

**Result:**

Application of the ProMIS platform produced PMN310, a clinical‐stage, humanized monoclonal antibody highly selective for Abeta oligomers without significant reactivity with Abeta monomers or fibrils, thereby avoiding target distraction by these more abundant species, and reducing the risk of brain edema and microhemorrhages associated with the targeting of vascular/parenchymal amyloid. Similarly, specific epitopes for alpha‐synuclein toxic oligomers/soluble fibrils that drive synucleinopathies, and for pathogenic TDP‐43 in ALS/FTD have been identified and lead candidate antibodies generated. The small size and precise conformation of these epitopes have been translated into vaccines, enabling the specific targeting of pathogenic molecular species in preclinical models.

**Conclusion:**

ProMIS has circumvented the specificity limitations of conventional immunizations to enable selective passive and active immunotherapies for neurodegenerative diseases.

